# Supplementary material for: Validation of a molecular workflow for Cochliomyia hominivorax (New World screwworm) identification in field samples
Source: Sci Rep. 2026 Jun 3;16:25283. doi: 10.1038/s41598-026-54866-6 (PMC13469346; doi:10.1038/s41598-026-54866-6)
Supplement: Supplementary file 2 [file 41598_2026_54866_MOESM2_ESM.pdf]

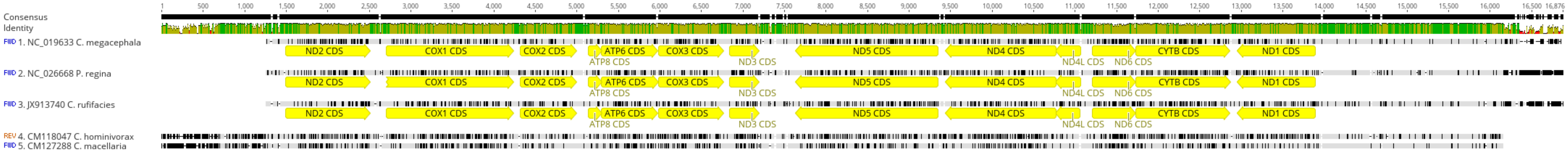

Supplemental Figure 1. Mitochondrial Alignment. Alignment of mitochrondrial sequence from *C. megacephala, P. regina, C. rufifacies, C. hominivorax, and C. macellaria*

Supplemental Table 1. Sample Metadata

Tube Number	Species	Sex	Accession	Collection Date	Collection State	Collection County
1	Lucilia sp.	F	25P00371	11/24/2025	California	San Diego
2	Cochliomyia macellaria	F	25P00371	11/24/2025	California	San Diego
3	Sarcophagidae		25P00374	11/24/2025	California	Imperial
4	Cochliomyia macellaria	M	25P00374	11/24/2025	California	Imperial
5	Cochliomyia macellaria	F	25P00374	11/24/2025	California	Imperial
6	Chrysomya megacephala	M	25P00370	11/24/2025	Texas	Hidalgo
7	Chrysomya megacephala	F	25P00370	11/24/2025	Texas	Hidalgo
8	Chrysomya rufifacies	F	25P00370	11/24/2025	Texas	Hidalgo
9	Chrysomya rufifacies	F	25P00370	11/24/2025	Texas	Hidalgo
10	Chrysomya megacephala	F	25P00370	11/24/2025	Texas	Hidalgo
11	Cochliomyia hominivorax	F	NWS - COPEG			
12	Chrysomya megacephala	F	25P00370	11/24/2025	Texas	Hidalgo
13	Cochliomyia macellaria	M	25P00370	11/24/2025	Texas	Hidalgo
14	Chrysomya rufifacies	M	25P00370	11/24/2025	Texas	Hidalgo
15	Cochliomyia macellaria	F	25P00365	11/24/2025	Texas	Cameron
16	Chrysomya rufifacies	M	25P00365	11/24/2025	Texas	Cameron
17	Cochliomyia hominivorax	M	NWS - COPEG			
18	Composomyiops callipes	F	ARS - Colony			
19	Cochliomyia macellaria	F	25P00390	12/1/2025	Texas	Starr
20			25P00390	12/1/2025	Texas	Starr
21			25P00390	12/1/2025	Texas	Starr
22	Chrysomya rufifacies	F	25P00390	12/1/2025	Texas	Starr
23	Chrysomya rufifacies	F	25P00390	12/1/2025	Texas	Starr
24	Cochliomyia macellaria	F	25P00390	12/1/2025	Texas	Starr
25	Chrysomya rufifacies	F	25P00390	12/1/2025	Texas	Starr
26			25P00390	12/1/2025	Texas	Starr
27	Cochliomyia macellaria	F	25P00390	12/1/2025	Texas	Starr
28	Chrysomya megacephala	M	25P00390	12/1/2025	Texas	Starr
29	Chrysomya rufifacies	M	25P00390	12/1/2025	Texas	Starr
30	Cochliomyia macellaria	F	25P00390	12/1/2025	Texas	Starr
31	Chrysomya rufifacies	F	25P00390	12/1/2025	Texas	Starr
32	Cochliomyia macellaria	F	25P00402	12/1/2025	Texas	Zapata
33	Cochliomyia macellaria	M	25P00402	12/1/2025	Texas	Zapata
34			25P00402	12/1/2025	Texas	Zapata
35	Cochliomyia macellaria	F	25P00402	12/1/2025	Texas	Zapata
36	Chrysomya rufifacies	F	25P00402	12/1/2025	Texas	Zapata
37	Cochliomyia macellaria	F	25P00402	12/1/2025	Texas	Zapata
38	Chrysomya rufifacies	F	25P00402	12/1/2025	Texas	Zapata
39	Cochliomyia macellaria	F	25P00402	12/1/2025	Texas	Zapata
40	Cochliomyia macellaria	F	25P00402	12/1/2025	Texas	Zapata
41	Cochliomyia macellaria	M	25P00402	12/1/2025	Texas	Zapata
42			25P00402	12/1/2025	Texas	Zapata
43	Cochliomyia macellaria	M	25P00402	12/1/2025	Texas	Zapata
44	Chrysomya rufifacies	F	25P00414	12/4/2025	Texas	Val Verde
45	Cochliomyia macellaria	M	25P00414	12/4/2025	Texas	Val Verde
46	Chrysomya rufifacies	F	25P00414	12/4/2025	Texas	Val Verde

47	Cochliomyia macellaria	M	25P00414	12/4/2025	Texas	Val Verde
48			25P00414	12/4/2025	Texas	Val Verde
49	Cochliomyia macellaria	F	25P00414	12/4/2025	Texas	Val Verde
50	Chrysomya rufifacies	M	25P00414	12/4/2025	Texas	Val Verde
51	Cochliomyia macellaria	F	25P00418	12/4/2025	Texas	Maverick/Webb
52	Cochliomyia macellaria	F	25P00418	12/4/2025	Texas	Maverick/Webb
53	Cochliomyia macellaria	F	25P00418	12/4/2025	Texas	Maverick/Webb
54	Phormia regina	M	25P00418	12/4/2025	Texas	Maverick/Webb
55	Cochliomyia macellaria	F	25P00418	12/4/2025	Texas	Maverick/Webb
56	Phormia regina	F	25P00418	12/4/2025	Texas	Maverick/Webb
57	Cochliomyia macellaria	M	25P00417	12/4/2025	Texas	Zapata
58	Cochliomyia macellaria	F	25P00417	12/4/2025	Texas	Zapata
59	Chrysomya megacephala	M	25P00417	12/4/2025	Texas	Zapata
60	Chrysomya rufifacies	M	25P00419	12/4/2025	Arizona	Cochise
61	Chrysomya rufifacies	M	25P00419	12/4/2025	Arizona	Cochise
62	Chrysomya rufifacies	M	25P00419	12/4/2025	Arizona	Cochise
63	Chrysomya megacephala	F	25P00419	12/4/2025	Arizona	Cochise
64	Cochliomyia macellaria	M	25P00458	12/4/2025	California	San Diego
65	Cochliomyia macellaria	M	25P00458	12/4/2025	California	San Diego
66	Cochliomyia macellaria	F	25P00458	12/4/2025	California	San Diego
67	Cochliomyia macellaria	F	25P00458	12/4/2025	California	San Diego
68	Cochliomyia macellaria	M	25P00458	12/4/2025	California	San Diego
69	Cochliomyia macellaria	M	25P00458	12/4/2025	California	San Diego
70	Cochliomyia macellaria	F	25P00458	12/4/2025	California	San Diego
71	Cochliomyia macellaria	M	25P00458	12/4/2025	California	San Diego
72	Cochliomyia macellaria	F	25P00458	12/4/2025	California	San Diego
73	Cochliomyia macellaria	F	25P00458	12/4/2025	California	San Diego
74	Cochliomyia macellaria	M	25P00458	12/4/2025	California	San Diego
75	Cochliomyia macellaria	F	25P00458	12/4/2025	California	San Diego
76	Cochliomyia macellaria	F	25P00458	12/4/2025	California	San Diego
77	Cochliomyia macellaria	M	25P00458	12/4/2025	California	San Diego
78	Cochliomyia hominivorax		NWS - COPEG			
79	Cochliomyia macellaria	M	25P00458	12/4/2025	California	San Diego
80	Cochliomyia macellaria	M	25P00458	12/4/2025	California	San Diego
81	Cochliomyia macellaria	F	25P00458	12/4/2025	California	San Diego
82	Cochliomyia macellaria	F	25P00457	12/4/2025	Texas	Starr
83	Chrysomya rufifacies	F	25P00457	12/4/2025	Texas	Starr
84	Chrysomya rufifacies	F	25P00457	12/4/2025	Texas	Starr
85			25P00457	12/4/2025	Texas	Starr
86	Chrysomya rufifacies	F	25P00457	12/4/2025	Texas	Starr
87	Cochliomyia macellaria	F	25P00457	12/4/2025	Texas	Starr
88	Chrysomya rufifacies	M	25P00457	12/4/2025	Texas	Starr
89	Chrysomya rufifacies	F	25P00457	12/4/2025	Texas	Starr
90	Cochliomyia macellaria	M	25P00457	12/4/2025	Texas	Starr
91			25P00457	12/4/2025	Texas	Starr
92	Cochliomyia macellaria	M	25P00457	12/4/2025	Texas	Starr
93	Chrysomya rufifacies	M	25P00457	12/4/2025	Texas	Starr
94			25P00457	12/4/2025	Texas	Starr
95	Chrysomya rufifacies	F	25P00457	12/4/2025	Texas	Starr
96	Cochliomyia hominivorax		NWS - COPEG			

97	Cochliomyia macellaria	F	25P00385	11/26/2025	Texas	Cameron
98	Cochliomyia macellaria	F	25P00385	11/26/2025	Texas	Cameron
99	Cochliomyia macellaria	M	25P00385	11/26/2025	Texas	Cameron
100	Cochliomyia macellaria	F	25P00385	11/26/2025	Texas	Cameron
101	Chrysomya rufifacies	F	25P00385	11/26/2025	Texas	Cameron
102	Cochliomyia macellaria	F	25P00385	11/26/2025	Texas	Cameron
103			25P00385	11/26/2025	Texas	Cameron
104	Cochliomyia macellaria	F	25P00385	11/26/2025	Texas	Cameron
105	Chrysomya rufifacies	F	25P00385	11/26/2025	Texas	Cameron
106	Cochliomyia macellaria	F	25P00385	11/26/2025	Texas	Cameron
107	Cochliomyia macellaria	M	25P00385	11/26/2025	Texas	Cameron
108	Cochliomyia macellaria	F	25P00385	11/26/2025	Texas	Cameron
109	Angioneur sp.	F	25P00367	11/24/2025	Texas	Val Verde
110	Chrysomya rufifacies	M	25P00367	11/24/2025	Texas	Val Verde
111	Chrysomya rufifacies	F	25P00367	11/24/2025	Texas	Val Verde
112			25P00367	11/24/2025	Texas	Val Verde
113	Chrysomya rufifacies	M	25P00367	11/24/2025	Texas	Val Verde
114	Chrysomya rufifacies	M	25P00367	11/24/2025	Texas	Val Verde
115	Cochliomyia macellaria	F	25P00399	12/1/2025	California	Imperial
116	Cochliomyia macellaria	F	25P00399	12/1/2025	California	Imperial
117	Cochliomyia macellaria	F	25P00399	12/1/2025	California	Imperial
118	Cochliomyia hominivorax	M	NWS - COPEG			
119	Cochliomyia macellaria	F	25P00399	12/1/2025	California	Imperial
120	Cochliomyia macellaria	M	25P00399	12/1/2025	California	Imperial
121	Cochliomyia macellaria	F	25P00399	12/1/2025	California	Imperial
122	Cochliomyia macellaria	F	25P00399	12/1/2025	California	Imperial
123	Cochliomyia macellaria	M	25P00399	12/1/2025	California	Imperial
124	Cochliomyia macellaria	F	25P00399	12/1/2025	California	Imperial
125	Cochliomyia macellaria	F	25P00399	12/1/2025	California	Imperial
126	Cochliomyia macellaria	F	25P00399	12/1/2025	California	Imperial
127	Cochliomyia hominivorax	M	NWS - COPEG			
128	Cochliomyia macellaria	F	25P00399	12/1/2025	California	Imperial
129	Chrysomya megacephala	M	25P00393	12/1/2025	Texas	Hidalgo
130	Chrysomya megacephala	M	25P00389	11/28/2025	Texas	Hidalgo
131	Chrysomya megacephala	M	25P00389	11/28/2025	Texas	Hidalgo
132	Chrysomya rufifacies	M	25P00405	12/1/2025	Texas	Val Verde
133	Chrysomya rufifacies	M	25P00405	12/1/2025	Texas	Val Verde
134	Chrysomya rufifacies	M	25P00405	12/1/2025	Texas	Val Verde
135	Chrysomya rufifacies	M	25P00405	12/1/2025	Texas	Val Verde
136	Chrysomya rufifacies	M	25P00405	12/1/2025	Texas	Val Verde
137	Chrysomya rufifacies	M	25P00405	12/1/2025	Texas	Val Verde
138	Cochliomyia macellaria	M	25P00405	12/1/2025	Texas	Val Verde
139	Chrysomya rufifacies	M	25P00405	12/1/2025	Texas	Val Verde
140	Cochliomyia macellaria	F	25P00405	12/1/2025	Texas	Val Verde
141	Phormia regina	F	25P00405	12/1/2025	Texas	Val Verde
142	Cochliomyia macellaria	F	25P00405	12/1/2025	Texas	Val Verde
143	Cochliomyia macellaria	M	25P00405	12/1/2025	Texas	Val Verde
144	Cochliomyia macellaria	F	25P00405	12/1/2025	Texas	Val Verde
145	Chrysomya rufifacies	M	25P00404	12/2/2025	Arizona	Cochiese
146	Chrysomya rufifacies	M	25P00404	12/2/2025	Arizona	Cochiese

147	Chrysomya rufifacies	F	25P00404	12/2/2025	Arizona	Cochiese
148	Chrysomya megacephala	M	25P00404	12/2/2025	Arizona	Cochiese
149	Chrysomya megacephala	M	25P00404	12/2/2025	Arizona	Cochiese
150	Chrysomya megacephala	M	25P00404	12/2/2025	Arizona	Cochiese
151	Cochliomyia hominivorax	F	NWS - COPEG			
152	Cochliomyia macellaria	M	25P00404	12/2/2025	Arizona	Cochise
153	Cochliomyia macellaria	M	25P00404	12/2/2025	Arizona	Cochise
154	Chrysomya rufifacies	F	25P00404	12/2/2025	Arizona	Cochise
155	Chrysomya rufifacies	M	25P00404	12/2/2025	Arizona	Cochise
156	Chrysomya rufifacies	F	25P00404	12/2/2025	Arizona	Cochise
157	Chrysomya megacephala	F	25P00404	12/2/2025	Arizona	Cochise
158	Chrysomya rufifacies	M	25P00455	12/4/2025	Texas	Webb
159			25P00455	12/4/2025	Texas	Webb
160	Phormia regina	M	25P00455	12/4/2025	Texas	Webb
161	Cochliomyia macellaria	M	25P00455	12/4/2025	Texas	Webb
162	Cochliomyia macellaria	M	25P00455	12/4/2025	Texas	Webb
163	Chrysomya rufifacies	M	25P00455	12/4/2025	Texas	Webb
164	Chrysomya rufifacies	M	25P00455	12/4/2025	Texas	Webb
165			25P00455	12/4/2025	Texas	Webb
166	Chrysomya rufifacies	M	25P00455	12/4/2025	Texas	Webb
167	Cochliomyia macellaria	F	25P00455	12/4/2025	Texas	Webb
168	Cochliomyia macellaria	M	25P00455	12/4/2025	Texas	Webb
169	Chrysomya megacephala	F	25P00455	12/4/2025	Texas	Webb
170			25P00455	12/4/2025	Texas	Webb
171	Cochliomyia macellaria	F	25P00455	12/4/2025	Texas	Webb
172	Chrysomya megacephala	F	25P00456	12/4/2025	Texas	Hidalgo
173	Cochliomyia macellaria	M	25P00456	12/4/2025	Texas	Hidalgo
174	Sarcophagidae	F	25P00456	12/4/2025	Texas	Hidalgo
175	Cochliomyia macellaria	M	25P00456	12/4/2025	Texas	Hidalgo
176	Sarcophagidae	F	25P00456	12/4/2025	Texas	Hidalgo
177	Chrysomya rufifacies	M	25P00456	12/4/2025	Texas	Hidalgo
178	Chrysomya rufifacies	M	25P00456	12/4/2025	Texas	Hidalgo
179	Chrysomya megacephala	M	25P00456	12/4/2025	Texas	Hidalgo
180			25P00456	12/4/2025	Texas	Hidalgo
181	Cochliomyia hominivorax		NWS - COPEG	12/4/2025	Texas	Hidalgo
182			25P00456	12/4/2025	Texas	Hidalgo
183	Chrysomya rufifacies	F	25P00456	12/4/2025	Texas	Hidalgo
184	Cochliomyia macellaria	M	25P00456	12/4/2025	Texas	Hidalgo
185	Chrysomya rufifacies	M	25P00456	12/4/2025	Texas	Hidalgo
186	Cochliomyia macellaria	M	25P00456	12/4/2025	Texas	Hidalgo
187	Cochliomyia macellaria	F	25P00456	12/4/2025	Texas	Hidalgo
188	Chrysomya megacephala	M	25P00456	12/4/2025	Texas	Hidalgo
189	Cochliomyia macellaria	F	25P00456	12/4/2025	Texas	Hidalgo
190	Cochliomyia macellaria	F	25P00456	12/4/2025	Texas	Hidalgo
191	Cochliomyia macellaria	F	25P00456	12/4/2025	Texas	Hidalgo
192	Cochliomyia macellaria	F	25P00456	12/4/2025	Texas	Hidalgo
193	Cochliomyia macellaria	M	25P00456	12/4/2025	Texas	Hidalgo
194	Cochliomyia macellaria	M	25P00456	12/4/2025	Texas	Hidalgo
195	Cochliomyia macellaria	M	25P00456	12/4/2025	Texas	Hidalgo
196	Cochliomyia macellaria	F	25P00456	12/4/2025	Texas	Hidalgo

197	Chrysomya rufifacies	M	25P00456	12/4/2025	Texas	Hidalgo
198	Chrysomya rufifacies	M	25P00456	12/4/2025	Texas	Hidalgo
199	Chrysomya rufifacies	M	25P00456	12/4/2025	Texas	Hidalgo
200	Chrysomya rufifacies	M	25P00456	12/4/2025	Texas	Hidalgo
201	Cochliomyia macellaria	F	25P00456	12/4/2025	Texas	Hidalgo
202	Chrysomya megacephala	M	25P00456	12/4/2025	Texas	Hidalgo
203	Chrysomya rufifacies	F	25P00456	12/4/2025	Texas	Hidalgo
204	Cochliomyia macellaria	M	25P00456	12/4/2025	Texas	Hidalgo
205	Chrysomya rufifacies	F	25P00456	12/4/2025	Texas	Hidalgo
206	Chrysomya megacephala	M	25P00456	12/4/2025	Texas	Hidalgo
207	Cochliomyia macellaria	M	25P00456	12/4/2025	Texas	Hidalgo
208	Cochliomyia hominivorax		NWS - COPEG			
209	Cochliomyia macellaria	F	25P00458	12/4/2025	California	Arizona
210	Cochliomyia macellaria	M	25P00458	12/4/2025	California	Arizona
211	Cochliomyia macellaria	M	25P00458	12/4/2025	California	Arizona
212	Chrysomya rufifacies	M	25P00458	12/4/2025	California	Arizona
213	Chrysomya rufifacies	M	25P00458	12/4/2025	California	Arizona
214			25P00458	12/4/2025	California	Arizona
215	Chrysomya rufifacies	M	25P00458	12/4/2025	California	Arizona
216	Chrysomya rufifacies	M	25P00458	12/4/2025	California	Arizona
217	Cochliomyia macellaria	M	25P00458	12/4/2025	California	Arizona
218	Mix 1					
219	Cochliomyia macellaria	F	25P00458	12/4/2025	California	Arizona
220	Cochliomyia macellaria	F	25P00458	12/4/2025	California	Arizona
221	Cochliomyia macellaria	F	25P00458	12/4/2025	California	Arizona
222	Cochliomyia macellaria	M	25P00458	12/4/2025	California	Arizona
223	Cochliomyia macellaria	M	25P00458	12/4/2025	California	Arizona
224	Cochliomyia macellaria	M	25P00458	12/4/2025	California	Arizona
225	Cochliomyia macellaria	F	25P00458	12/4/2025	California	Arizona
226	Cochliomyia macellaria	M	25P00458	12/4/2025	California	Arizona
227	Chrysomya rufifacies	F	25P00458	12/4/2025	California	Arizona
228	Cochliomyia macellaria	M	25P00458	12/4/2025	California	Arizona
229	Cochliomyia macellaria	F	25P00458	12/4/2025	California	Arizona
230	Cochliomyia macellaria	F	25P00458	12/4/2025	California	Arizona
231	Lucilia sp.	F	25P00528	12/11/2025	New Mexico	Luna
232	Chrysomya rufifacies	F	25P00528	12/11/2025	New Mexico	Luna
233	Cochliomyia macellaria	F	25P00528	12/11/2025	New Mexico	Luna
234	Chrysomya rufifacies	M	25P00541	12/15/2025	New Mexico	Luna
235	Chrysomya rufifacies	M	25P00541	12/15/2025	New Mexico	Luna
236	Cochliomyia macellaria	F	25P00529	12/11/2025	California	San Diego
237	Cochliomyia macellaria	M	25P00529	12/11/2025	California	San Diego
238	Cochliomyia macellaria	M	25P00529	12/11/2025	California	San Diego
239	Cochliomyia hominivorax		NWS - COPEG			
240						
241	Cochliomyia macellaria	M	25P00529	12/11/2025	California	San Diego
242	Cochliomyia macellaria	F	25P00529	12/11/2025	California	San Diego
243	Cochliomyia macellaria	M	25P00529	12/11/2025	California	San Diego
244						
245	Sarcophagidae	M	25P00527	12/11/2025	Arizona	Cochise
246			25P00527	12/11/2025	Arizona	Cochise

247	Chrysomya rufifacies	M	25P00527	12/11/2025	Arizona	Cochise
248	Cochliomyia macellaria	F	25P00539	12/11/2025	California	Imperial
249	Cochliomyia macellaria	F	25P00539	12/11/2025	California	Imperial
250	Mix 2					
251	Cochliomyia macellaria	F	25P00539	12/11/2025	California	Imperial
252	Chrysomya rufifacies	F	25P00539	12/11/2025	California	Imperial
253	Cochliomyia macellaria	F	25P00525	12/8/2025	California	San Diego
254	Cochliomyia macellaria	F	25P00525	12/8/2025	California	San Diego
255	Cochliomyia macellaria	F	25P00525	12/8/2025	California	San Diego
256	Cochliomyia macellaria	F	25P00525	12/8/2025	California	San Diego
257	Cochliomyia macellaria	F	25P00525	12/8/2025	California	San Diego
258	Cochliomyia macellaria	F	25P00525	12/8/2025	California	San Diego
259	Cochliomyia macellaria	F	25P00554	12/15/2025	California	Imperial
260	Cochliomyia macellaria	F	25P00554	12/15/2025	California	Imperial
261	Cochliomyia macellaria	F	25P00554	12/15/2025	California	Imperial
262	Cochliomyia macellaria	F	25P00554	12/15/2025	California	Imperial
263	Cochliomyia macellaria	F	25P00554	12/15/2025	California	Imperial
264	Cochliomyia macellaria	M	25P00554	12/15/2025	California	Imperial
265	Cochliomyia macellaria	F	25P00554	12/15/2025	California	Imperial
266	Cochliomyia macellaria	M	25P00554	12/15/2025	California	Imperial
267	Cochliomyia macellaria	F	25P00554	12/15/2025	California	Imperial
268	Cochliomyia macellaria	F	25P00554	12/15/2025	California	Imperial
269	Cochliomyia macellaria	F	25P00491	12/8/2025	Texas	Cameron
270	Cochliomyia macellaria	F	25P00491	12/8/2025	Texas	Cameron
271	Cochliomyia macellaria	M	25P00491	12/8/2025	Texas	Cameron
272	Chrysomya megacephala	M	25P00491	12/8/2025	Texas	Cameron
273	Chrysomya rufifacies	M	25P00491	12/8/2025	Texas	Cameron
274	Sarcophagidae		25P00491	12/8/2025	Texas	Cameron
275	Cochliomyia macellaria	M	25P00491	12/8/2025	Texas	Cameron
276	Cochliomyia hominivorax		25P00491	12/8/2025	Texas	Cameron
277	Cochliomyia macellaria	M	25P00491	12/8/2025	Texas	Cameron
278	Cochliomyia macellaria	M	25P00491	12/8/2025	Texas	Cameron
279	Mix 3					
280	Cochliomyia macellaria	M	25P00491	12/8/2025	Texas	Cameron
281	Cochliomyia macellaria	F	25P00484	12/8/2025	California	Imperial
282	Cochliomyia macellaria	F	25P00484	12/8/2025	California	Imperial
283			25P00484	12/8/2025	California	Imperial
284	Cochliomyia macellaria	F	25P00484	12/8/2025	California	Imperial
285	Cochliomyia macellaria	F	25P00484	12/8/2025	California	Imperial
286	Sarcophagidae		25P00484	12/8/2025	California	Imperial
287	Cochliomyia macellaria	F	25P00484	12/8/2025	California	Imperial
288	Cochliomyia macellaria	F	25P00484	12/8/2025	California	Imperial
289	Cochliomyia hominivorax					
290	Cochliomyia hominivorax					
291	Chrysomya rufifacies	F	25P00458	12/4/2025	California	San Diego
292	Cochliomyia macellaria	M	25P00539	12/11/2025	California	Imperial
293	Cochliomyia macellaria	F	25P00491	12/8/2025	Texas	Cameron
294	Chrysomya megacephala	M	25P00518	* No collection date, received at NVSL 12/9/25	Texas	Starr
295	Cochliomyia macellaria	F	25P00518		Texas	Starr

296	Sarcophagidae		25P00518		Texas	Starr
297	Sarcophagidae		25P00518		Texas	Starr
298	Chrysomya rufifacies	M	25P00518		Texas	Starr
299	Chrysomya rufifacies	F	25P00518		Texas	Starr
300	Cochliomyia macellaria	F	25P00490	12/8/2025	Texas	Maverick/Webb
301	Mix 4					
302	Phormia regina	M	25P00490	12/8/2025	Texas	Maverick/Webb
303	Cochliomyia macellaria	M	25P00490	12/8/2025	Texas	Maverick/Webb

**Count of Species**	**Sex**			
Species	*F*	*M*	*(blank)*	**Grand Total**
Cochliomyia macellaria	95	60		155
Chrysomya rufifacies	29	42		71
Chrysomya megacephala	7	15		22
Cochliomyia hominivorax	2	3	8	13
Sarcophagidae	2	1	5	8
Phormia regina	2	3		5
Lucilia sp.	2			2
Mix 1			1	1
Angioneur sp.	1			1
Composomyiops callipes	1			1
Mix 2			1	1
Mix 3			1	1
Mix 4			1	1
(blank)				
Grand Total	141	124	17	282

Whole genome sequences

Species	Isolate Name	Isolate Source	Date Collected	Collection Location	SRA Accession
Cochliomyia hominivorax	Belize-Maggot-402025	Not Collected	Not Collected	Belize	SRR37579083
Cochliomyia hominivorax	Female-Fertile-NWS-fly-251212	COPEG fertile specimen	12/12/2025	Panama	SRR37579082
Cochliomyia hominivorax	Female-Fertile-NWS-fly-251114	COPEG fertile specimen	11/14/2025	Panama	SRR37579071
Cochliomyia hominivorax	Female-Fertile-NWS-fly-251121	COPEG fertile specimen	11/21/2025	Panama	SRR37579061
Cochliomyia hominivorax	Female-Fertile-NWS-fly-251205	COPEG fertile specimen	12/5/2025	Panama	SRR37579060
Cochliomyia hominivorax	Belize-Maggot-417	Not Collected	Not Collected	Belize	SRR37579059
Cochliomyia hominivorax	Male-Fertile-NWS-fly-251205	COPEG fertile specimen	12/5/2025	Panama	SRR37579058
Cochliomyia hominivorax	Male-Fertile-NWS-fly-251114	COPEG fertile specimen	11/14/2025	Panama	SRR37579057
Cochliomyia hominivorax	Panama-Maggot	Not Collected	Not Collected	Panama	SRR37579056
Cochliomyia hominivorax	Female-Fertile-NWS-fly-251219	Not Collected	12/19/2025	Panama	SRR37579055
Cochliomyia hominivorax	Female-Sterile-NWS-fly-251128	COPEG sterile specimen	11/28/2025	Panama	SRR37579081
Cochliomyia hominivorax	Female-Fertile-NWS-fly-251128	COPEG fertile specimen	11/28/2025	Panama	SRR37579080
Cochliomyia hominivorax	Female-Sterile-NWS-fly-251121	COPEG sterile specimen	12/21/2025	Panama	SRR37579079
Cochliomyia hominivorax	Female-Sterile-NWS-fly-251212	COPEG sterile specimen	12/12/2025	Panama	SRR37579078
Cochliomyia hominivorax	Male-Sterile-NWS-fly-251205	COPEG sterile specimen	12/5/2025	Panama	SRR37579077
Cochliomyia hominivorax	Male-Fertile-NWS-fly-251128	COPEG fertile specimen	11/28/2025	Panama	SRR37579076
Cochliomyia hominivorax	Male-Sterile-NWS-fly-251219	COPEG sterile specimen	12/19/2025	Panama	SRR37579075
Cochliomyia hominivorax	Male-Fertile-NWS-fly-251212	COPEG fertile specimen	12/12/2025	Panama	SRR37579074
Cochliomyia hominivorax	Male-Fertile-NWS-fly-251121	COPEG fertile specimen	11/21/2025	Panama	SRR37579073

Cochliomyia hominivorax	Male-Sterile-NWS-fly-251128	COPEG sterile specimen	11/28/2025	Panama	SRR37579072
Cochliomyia hominivorax	Male-Sterile-NWS-fly-251114	COPEG sterile specimen	11/14/2025	Panama	SRR37579070
Cochliomyia hominivorax	Female-Sterile-NWS-fly-251219	COPEG sterile specimen	12/19/2025	Panama	SRR37579069
Cochliomyia hominivorax	Female-Sterile-NWS-fly-251205	COPEG sterile specimen	12/5/2025	Panama	SRR37579068
Cochliomyia hominivorax	Male-Sterile-NWS-fly-251212	COPEG sterile specimen	12/12/2025	Panama	SRR37579067
Cochliomyia hominivorax	Male-Sterile-NWS-fly-251121	COPEG sterile specimen	11/21/2025	Panama	SRR37579066
Cochliomyia hominivorax	Male-Fertile-NWS-fly-251219	COPEG fertile specimen	12/19/2025	Panama	SRR37579065
Cochliomyia hominivorax	Female-Sterile-NWS-fly-251114	COPEG sterile specimen	11/14/2025	Panama	SRR37579064
Cochliomyia hominivorax	Guatemala-Maggot	Not Collected	Not Collected	Guatemala	SRR37579063
Cochliomyia hominivorax	Mexico-Maggot	Not Collected	Not Collected	Mexico	SRR37579062
Cochliomyia macellaria	1-macellaria-PCRFree	Not Collected	Not Collected	Not Collected	SRR37600761
Cochliomyia macellaria	1-macellaria	Not Collected	Not Collected	Not Collected	SRR37600760
Chrysomya megacephala	2-meqacephala-PCRFree	Not Collected	Not Collected	Not Collected	SRR37600759
Chrysomya megacephala	2-meqacephala	Not Collected	Not Collected	Not Collected	SRR37600758
Chrysomya megacephala	3-meqacephala-PCRFree	Not Collected	Not Collected	Not Collected	SRR37600757
Chrysomya megacephala	3-meqacephala	Not Collected	Not Collected	Not Collected	SRR37600756
Chrysomya megacephala	4-meqacephala-PCRFree	Not Collected	Not Collected	Not Collected	SRR37600755
Chrysomya megacephala	4-meqacephala	Not Collected	Not Collected	Not Collected	SRR37600754
Phormia regina	5-regina-PCRFree	Not Collected	Not Collected	Not Collected	SRR37600753
Phormia regina	5-regina	Not Collected	Not Collected	Not Collected	SRR37600752

Sanger Amplicon Sequences

Cochliomyia hominivorax	Belize-Larva-1-Amplicon1	Not Collected	Not Collected	Belize	SRR37599751
Cochliomyia hominivorax	Belize-Larva-2-Amplicon1	Not Collected	Not Collected	Belize	SRR37599750
Cochliomyia hominivorax	Costa Rica-Larva-1-Amplicon1	Not Collected	Not Collected	Costa Rica	SRR37599655
Cochliomyia hominivorax	Costa Rica-Larva-2-Amplicon1	Not Collected	Not Collected	Costa Rica	SRR37599644
Cochliomyia hominivorax	Costa Rica-Larva-3-Amplicon1	Not Collected	Not Collected	Costa Rica	SRR37599689
Cochliomyia hominivorax	El Salvador-Larva-1-Amplicon1	Not Collected	Not Collected	El Salvador	SRR37599678
Cochliomyia hominivorax	El Salvador-Larva-2-Amplicon1	Not Collected	Not Collected	El Salvador	SRR37599667
Cochliomyia hominivorax	El Salvador-Larva-3-Amplicon1	Not Collected	Not Collected	El Salvador	SRR37599740
Cochliomyia hominivorax	Guatemala-Larva-1-Amplicon1	Not Collected	Not Collected	Guatemala	SRR37599729
Cochliomyia hominivorax	Guatemala-Larva-2-Amplicon1	Not Collected	Not Collected	Guatemala	SRR37599634
Cochliomyia hominivorax	Guatemala-Larva-3-Amplicon1	Not Collected	Not Collected	Guatemala	SRR37599749
Cochliomyia hominivorax	Honduras-Larva-1-Amplicon1	Not Collected	Not Collected	Honduras	SRR37599710
Cochliomyia hominivorax	Honduras-Larva-2-Amplicon1	Not Collected	Not Collected	Honduras	SRR37599699
Cochliomyia hominivorax	Honduras-Larva-3-Amplicon1	Not Collected	Not Collected	Honduras	SRR37599662
Cochliomyia hominivorax	Mexico-Larva-1-Amplicon1	Not Collected	Not Collected	Mexico	SRR37599661
Cochliomyia hominivorax	Mexico-Larva-2-Amplicon1	Not Collected	Not Collected	Mexico	SRR37599660
Cochliomyia hominivorax	Nicaragua-Larva-1-Amplicon1	Not Collected	Not Collected	Nicaragua	SRR37599659
Cochliomyia hominivorax	Nicaragua-Larva-2-Amplicon1	Not Collected	Not Collected	Nicaragua	SRR37599658
Cochliomyia hominivorax	Nicaragua-Larva-3-Amplicon1	Not Collected	Not Collected	Nicaragua	SRR37599657
Cochliomyia hominivorax	Panama-Larva-1-Amplicon1	Not Collected	Not Collected	Panama	SRR37599656
Cochliomyia hominivorax	Panama-Larva-2-Amplicon1	Not Collected	Not Collected	Panama	SRR37599654
Cochliomyia hominivorax	Panama-Larva-3-Amplicon1	Not Collected	Not Collected	Panama	SRR37599653
Cochliomyia hominivorax	COPEG-Larva-1-Amplicon1	Not Collected	Not Collected	Panama	SRR37599652
Cochliomyia hominivorax	COPEG-Larva-2-Amplicon1	Not Collected	Not Collected	Panama	SRR37599651
Cochliomyia hominivorax	COPEG-Larva-3-Amplicon1	Not Collected	Not Collected	Panama	SRR37599650
Cochliomyia hominivorax	COPEG-Larva-4-Amplicon1	Not Collected	Not Collected	Panama	SRR37599649
Cochliomyia hominivorax	COPEG-flyleg-11-Amplicon1	Not Collected	Not Collected	Panama	SRR37599648
Cochliomyia hominivorax	COPEG-flyleg-17-Amplicon1	Not Collected	Not Collected	Panama	SRR37599647

Cochliomyia hominivorax	COPEG-flyleg-96-Amplicon1	Not Collected	Not Collected	Panama	SRR37599646
Cochliomyia hominivorax	COPEG-flyleg-127-Amplicon1	Not Collected	Not Collected	Panama	SRR37599645
Cochliomyia hominivorax	COPEG-flyleg-151-Amplicon1	Not Collected	Not Collected	Panama	SRR37599643
Cochliomyia hominivorax	COPEG-flyleg-181-Amplicon1	Not Collected	Not Collected	Panama	SRR37599642
Cochliomyia hominivorax	Belize-Larva-1-Amplicon4	Not Collected	Not Collected	Belize	SRR37599641
Cochliomyia hominivorax	Belize-Larva-2-Amplicon4	Not Collected	Not Collected	Belize	SRR37599640
Cochliomyia hominivorax	Costa Rica-Larva-1-Amplicon4	Not Collected	Not Collected	Costa Rica	SRR37599639
Cochliomyia hominivorax	Costa Rica-Larva-2-Amplicon4	Not Collected	Not Collected	Costa Rica	SRR37599638
Cochliomyia hominivorax	Costa Rica-Larva-3-Amplicon4	Not Collected	Not Collected	Costa Rica	SRR37599637
Cochliomyia hominivorax	El Salvador-Larva-1-Amplicon4	Not Collected	Not Collected	El Salvador	SRR37599692
Cochliomyia hominivorax	El Salvador-Larva-2-Amplicon4	Not Collected	Not Collected	El Salvador	SRR37599691
Cochliomyia hominivorax	El Salvador-Larva-3-Amplicon4	Not Collected	Not Collected	El Salvador	SRR37599690
Cochliomyia hominivorax	Guatemala-Larva-1-Amplicon4	Not Collected	Not Collected	Guatemala	SRR37599688
Cochliomyia hominivorax	Guatemala-Larva-2-Amplicon4	Not Collected	Not Collected	Guatemala	SRR37599687
Cochliomyia hominivorax	Guatemala-Larva-3-Amplicon4	Not Collected	Not Collected	Guatemala	SRR37599686
Cochliomyia hominivorax	Honduras-Larva-1-Amplicon4	Not Collected	Not Collected	Honduras	SRR37599685
Cochliomyia hominivorax	Honduras-Larva-2-Amplicon4	Not Collected	Not Collected	Honduras	SRR37599684
Cochliomyia hominivorax	Honduras-Larva-3-Amplicon4	Not Collected	Not Collected	Honduras	SRR37599683
Cochliomyia hominivorax	Mexico-Larva-1-Amplicon4	Not Collected	Not Collected	Mexico	SRR37599682
Cochliomyia hominivorax	Mexico-Larva-2-Amplicon4	Not Collected	Not Collected	Mexico	SRR37599681
Cochliomyia hominivorax	Nicaragua-Larva-1-Amplicon4	Not Collected	Not Collected	Nicaragua	SRR37599680
Cochliomyia hominivorax	Nicaragua-Larva-2-Amplicon4	Not Collected	Not Collected	Nicaragua	SRR37599679
Cochliomyia hominivorax	Nicaragua-Larva-3-Amplicon4	Not Collected	Not Collected	Nicaragua	SRR37599677
Cochliomyia hominivorax	Panama-Larva-1-Amplicon4	Not Collected	Not Collected	Panama	SRR37599676
Cochliomyia hominivorax	Panama-Larva-2-Amplicon4	Not Collected	Not Collected	Panama	SRR37599675
Cochliomyia hominivorax	Panama-Larva-3-Amplicon4	Not Collected	Not Collected	Panama	SRR37599674
Cochliomyia hominivorax	COPEG-Larva-1-Amplicon4	Not Collected	Not Collected	Panama	SRR37599673
Cochliomyia hominivorax	COPEG-Larva-2-Amplicon4	Not Collected	Not Collected	Panama	SRR37599672
Cochliomyia hominivorax	COPEG-Larva-3-Amplicon4	Not Collected	Not Collected	Panama	SRR37599671
Cochliomyia hominivorax	COPEG-Larva-4-Amplicon4	Not Collected	Not Collected	Panama	SRR37599670
Cochliomyia hominivorax	COPEG-flyleg-11-Amplicon4	Not Collected	Not Collected	Panama	SRR37599669
Cochliomyia hominivorax	COPEG-flyleg-17-Amplicon4	Not Collected	Not Collected	Panama	SRR37599668
Cochliomyia hominivorax	COPEG-flyleg-96-Amplicon4	Not Collected	Not Collected	Panama	SRR37599666
Cochliomyia hominivorax	COPEG-flyleg-127-Amplicon4	Not Collected	Not Collected	Panama	SRR37599665
Cochliomyia hominivorax	COPEG-flyleg-151-Amplicon4	Not Collected	Not Collected	Panama	SRR37599748
Cochliomyia hominivorax	COPEG-flyleg-181-Amplicon4	Not Collected	Not Collected	Panama	SRR37599747
Cochliomyia hominivorax	Belize-Larva-1-Amplicon5	Not Collected	Not Collected	Belize	SRR37599746
Cochliomyia hominivorax	Belize-Larva-2-Amplicon5	Not Collected	Not Collected	Belize	SRR37599745
Cochliomyia hominivorax	Costa Rica-Larva-1-Amplicon5	Not Collected	Not Collected	Costa Rica	SRR37599744
Cochliomyia hominivorax	Costa Rica-Larva-2-Amplicon5	Not Collected	Not Collected	Costa Rica	SRR37599743
Cochliomyia hominivorax	Costa Rica-Larva-3-Amplicon5	Not Collected	Not Collected	Costa Rica	SRR37599742
Cochliomyia hominivorax	El Salvador-Larva-1-Amplicon5	Not Collected	Not Collected	El Salvador	SRR37599741
Cochliomyia hominivorax	El Salvador-Larva-2-Amplicon5	Not Collected	Not Collected	El Salvador	SRR37599739
Cochliomyia hominivorax	El Salvador-Larva-3-Amplicon5	Not Collected	Not Collected	El Salvador	SRR37599738
Cochliomyia hominivorax	Guatemala-Larva-1-Amplicon5	Not Collected	Not Collected	Guatemala	SRR37599737
Cochliomyia hominivorax	Guatemala-Larva-2-Amplicon5	Not Collected	Not Collected	Guatemala	SRR37599736
Cochliomyia hominivorax	Guatemala-Larva-3-Amplicon5	Not Collected	Not Collected	Guatemala	SRR37599735
Cochliomyia hominivorax	Honduras-Larva-1-Amplicon5	Not Collected	Not Collected	Honduras	SRR37599734
Cochliomyia hominivorax	Honduras-Larva-2-Amplicon5	Not Collected	Not Collected	Honduras	SRR37599733
Cochliomyia hominivorax	Honduras-Larva-3-Amplicon5	Not Collected	Not Collected	Honduras	SRR37599732

Cochliomyia hominivorax	Mexico-Larva-1-Amplicon5	Not Collected	Not Collected	Mexico	SRR37599731
Cochliomyia hominivorax	Mexico-Larva-2-Amplicon5	Not Collected	Not Collected	Mexico	SRR37599730
Cochliomyia hominivorax	Nicaragua-Larva-1-Amplicon5	Not Collected	Not Collected	Nicaragua	SRR37599728
Cochliomyia hominivorax	Nicaragua-Larva-2-Amplicon5	Not Collected	Not Collected	Nicaragua	SRR37599727
Cochliomyia hominivorax	Nicaragua-Larva-3-Amplicon5	Not Collected	Not Collected	Nicaragua	SRR37599726
Cochliomyia hominivorax	Panama-Larva-1-Amplicon5	Not Collected	Not Collected	Panama	SRR37599725
Cochliomyia hominivorax	Panama-Larva-2-Amplicon5	Not Collected	Not Collected	Panama	SRR37599724
Cochliomyia hominivorax	Panama-Larva-3-Amplicon5	Not Collected	Not Collected	Panama	SRR37599723
Cochliomyia hominivorax	COPEG-Larva-1-Amplicon5	Not Collected	Not Collected	Panama	SRR37599722
Cochliomyia hominivorax	COPEG-Larva-2-Amplicon5	Not Collected	Not Collected	Panama	SRR37599721
Cochliomyia hominivorax	COPEG-Larva-3-Amplicon5	Not Collected	Not Collected	Panama	SRR37599636
Cochliomyia hominivorax	COPEG-Larva-4-Amplicon5	Not Collected	Not Collected	Panama	SRR37599635
Cochliomyia hominivorax	COPEG-flyleg-11-Amplicon5	Not Collected	Not Collected	Panama	SRR37599633
Cochliomyia hominivorax	COPEG-flyleg-17-Amplicon5	Not Collected	Not Collected	Panama	SRR37599632
Cochliomyia hominivorax	COPEG-flyleg-96-Amplicon5	Not Collected	Not Collected	Panama	SRR37599631
Cochliomyia hominivorax	COPEG-flyleg-127-Amplicon5	Not Collected	Not Collected	Panama	SRR37599630
Cochliomyia hominivorax	COPEG-flyleg-151-Amplicon5	Not Collected	Not Collected	Panama	SRR37599629
Cochliomyia hominivorax	COPEG-flyleg-181-Amplicon5	Not Collected	Not Collected	Panama	SRR37599628
Cochliomyia hominivorax	Belize-Larva-1-Amplicon10	Not Collected	Not Collected	Belize	SRR37599627
Cochliomyia hominivorax	Belize-Larva-2-Amplicon10	Not Collected	Not Collected	Belize	SRR37599626
Cochliomyia hominivorax	Costa Rica-Larva-1-Amplicon10	Not Collected	Not Collected	Costa Rica	SRR37599625
Cochliomyia hominivorax	Costa Rica-Larva-2-Amplicon10	Not Collected	Not Collected	Costa Rica	SRR37599624
Cochliomyia hominivorax	Costa Rica-Larva-3-Amplicon10	Not Collected	Not Collected	Costa Rica	SRR37599720
Cochliomyia hominivorax	El Salvador-Larva-1-Amplicon10	Not Collected	Not Collected	El Salvador	SRR37599719
Cochliomyia hominivorax	El Salvador-Larva-2-Amplicon10	Not Collected	Not Collected	El Salvador	SRR37599718
Cochliomyia hominivorax	El Salvador-Larva-3-Amplicon10	Not Collected	Not Collected	El Salvador	SRR37599717
Cochliomyia hominivorax	Guatemala-Larva-1-Amplicon10	Not Collected	Not Collected	Guatemala	SRR37599716
Cochliomyia hominivorax	Guatemala-Larva-2-Amplicon10	Not Collected	Not Collected	Guatemala	SRR37599715
Cochliomyia hominivorax	Guatemala-Larva-3-Amplicon10	Not Collected	Not Collected	Guatemala	SRR37599714
Cochliomyia hominivorax	Honduras-Larva-1-Amplicon10	Not Collected	Not Collected	Honduras	SRR37599713
Cochliomyia hominivorax	Honduras-Larva-2-Amplicon10	Not Collected	Not Collected	Honduras	SRR37599712
Cochliomyia hominivorax	Honduras-Larva-3-Amplicon10	Not Collected	Not Collected	Honduras	SRR37599711
Cochliomyia hominivorax	Mexico-Larva-1-Amplicon10	Not Collected	Not Collected	Mexico	SRR37599709
Cochliomyia hominivorax	Mexico-Larva-2-Amplicon10	Not Collected	Not Collected	Mexico	SRR37599708
Cochliomyia hominivorax	Nicaragua-Larva-1-Amplicon10	Not Collected	Not Collected	Nicaragua	SRR37599707
Cochliomyia hominivorax	Nicaragua-Larva-2-Amplicon10	Not Collected	Not Collected	Nicaragua	SRR37599706
Cochliomyia hominivorax	Nicaragua-Larva-3-Amplicon10	Not Collected	Not Collected	Nicaragua	SRR37599705
Cochliomyia hominivorax	Panama-Larva-1-Amplicon10	Not Collected	Not Collected	Panama	SRR37599704
Cochliomyia hominivorax	Panama-Larva-2-Amplicon10	Not Collected	Not Collected	Panama	SRR37599703
Cochliomyia hominivorax	Panama-Larva-3-Amplicon10	Not Collected	Not Collected	Panama	SRR37599702
Cochliomyia hominivorax	COPEG-Larva-1-Amplicon10	Not Collected	Not Collected	Panama	SRR37599701
Cochliomyia hominivorax	COPEG-Larva-2-Amplicon10	Not Collected	Not Collected	Panama	SRR37599700
Cochliomyia hominivorax	COPEG-Larva-3-Amplicon10	Not Collected	Not Collected	Panama	SRR37599698
Cochliomyia hominivorax	COPEG-Larva-4-Amplicon10	Not Collected	Not Collected	Panama	SRR37599697
Cochliomyia hominivorax	COPEG-flyleg-11-Amplicon10	Not Collected	Not Collected	Panama	SRR37599696
Cochliomyia hominivorax	COPEG-flyleg-17-Amplicon10	Not Collected	Not Collected	Panama	SRR37599695
Cochliomyia hominivorax	COPEG-flyleg-96-Amplicon10	Not Collected	Not Collected	Panama	SRR37599694
Cochliomyia hominivorax	COPEG-flyleg-127-Amplicon10	Not Collected	Not Collected	Panama	SRR37599693
Cochliomyia hominivorax	COPEG-flyleg-151-Amplicon10	Not Collected	Not Collected	Panama	SRR37599664
Cochliomyia hominivorax	COPEG-flyleg-181-Amplicon10	Not Collected	Not Collected	Panama	SRR37599663

Supplemental Table 2. Previously Published Primers

**Torres 2004**	
Locus	Sequence
CH01	GCATTATACAGACTCAAACAC
	GTTTGTCTGGTTATAAGCTT
CH02	TCTTATAGCGCATAGACACG
	CATGAAGGCTGCTTGTATG
CH05	CAATCAACACAAACTCACG
	CACCGTTAGTATCGCATG
CH09	ACTATACCGGCCTCACAC
	TTGTTGTTGTATGAGTGGTG
CH10	TGTGGCAACATTTCTTTTG
	TTGGCAAAAAATGTCTCTG
CH11	TTGCTGCAACATACTGGG
	TAATTTGTTGGCGGCTTC
Ch12	ATGTCCATCACTTCAAGCAC
	TTTGCAGAGGAGTGAGCG
CH14	TGCTGTTGGGTGTTGTAG
	TTATGATGACCTTCTCTACG
CH15	ATCACAAGAGTAAACATTTC
	TGTGTGTGTAAGACATGC
CH20	ATCCATGTCCTACTCAGC
	TGCATTTCAATTGCTTTC

**Taylor et al., 1996**	
Sp2	CAGCTACTTTATGAGCTTTAGG
Sp3	CATTTCAAGCTGTGTAAGCATC
C2F3	GGTCAATGTTCAGAAATTTGTGG
Sp4	GAGACCATTACTTGCTTTCAGTCATCT
N4R2	GTAGGAGGAGCTGCTATATTAG
N4F3	GCTTATTCATCGGTTGCTCA
P6	ACATGAATTGGAGCTCGACCAGT
P7	GGTACATTACCTCGGTTTCGTTATGAT
P4	GGTCCCTTACGAATITGAATATATCCT
P5	GAGTTCAAACCGGCGTAAGCCAGGT

**Narang&Degrugillier, 1995**	
12sai	A CTA GGA TTA GAT ACC CTA TTA T
16sa	ATG TTT TTG ATAAAC AGGCG
16sar	CGCCTGTTTATCAAAAACAT
16sbr	CTCCGGTTTGAACTCAGATC
t0ISO	ATTTACCCTATCAAGGTAA
12sifi	CGGGCGATGTGTACATAATT
t-ISO	ATTTACCCTATCAAGGTAA
12sair	AGGGTATCTAATCCTAGTTT
TTGATTTTTTGGTCATCCAGAAGT	
GATCAATATCATTGACC	Hall and Smith 1991

**Taylor et al., 1996**	
Name	Sequence
SP1	TACAATTTATCGCCTAAACTTCAGCC
SP4	GAGACCATTACTTGCTTTCAGTCATCT
C2F3	GGTCAATGTTCAGAAATTTGTGG
N3R2	TGATTTCATTCATGATATAGTCC
N3F1	CCTTTTGAATGTGGATTTGATCC
N5F3	TTGTCTACTTTAAGACAATTAGG
P6	ACATGAATTGGAGCTCGACCAGT
P5	GAGTTCAAACCGGCGTAAGCCAGGT
Sp2	CAGCTACTTTATGAGCTTTAGG
SP3	CATTTCAAGCTGTGTAAGCATC
C3F1	ACAGGTGCTATTGGAGCTAT
C3R1	CCATAAACTGAATCAGCAAT
P6	
P7	GGTACATTACCTCGGTTTCGTTATGAT
P4	GGTCCCTTACGAATTTGAATATATCCT
P5	

**Simon et al., 1994**	
Name	Sequence
TI-N-8	CTATCAAGGTAACCCTTTTTATCAGGCA
TI-N-4	ATTTACCCTATCAAGGTAA
TM-J-206	GCTAATTAAGCTACTGGGTTCAT
TM-N-193	TGGGGTATGAACCCAGTAGC
N2-N-732	GAAGTTTGGTTTAAACCTCC
N2-J-748	ATTGGAGGTTTAAACCAAAC
N2-J-1006	TAGGTGGATTACCTCCATTTTTAGG
TY-J-1460	TACAATCTATCGCCTAAACTTCAGCC
C1-N-1560	TGTTCCTACTATTCCGGCTCA
C1-J-1718	GGGGGGTTTGGAAATTGATTAGTGCC
C1-J-1751	GGAGCTCCTGACATAGCATTCCC
C1-J-1859	GGTACAGGTTGAACTGTTTACCCTCC
C1-J-2183	CAACATTTATTTTGATTTTTTGG
C1-N-2191	CCCGGTAAAATTAAAATATAAACTTC
C1-J-2195	TTGATTTTTTGGTCACCCTGAAGT
C1-N-2329	ACTGTAAATATATGATGAGCTCA
C1-J-2441	CCTACAGGAATTAAAATTTTTAGATGATTAGC
C1-N-2659	GTCAATCCAGTAAATAATGG
C1-J-2797	CCTCGACGTTATTCAGATTACC
TL2-N-3014	TCCATTGCACTAATCTGCCATATTA
TL2-J-3037	ATGGCAGATTAGTGCAATGG
C2-J-3138	AGAGCTTCTCCTTTAATGGAACA
C2-J-3279	GGACAACTTATTGAAATAATTTGAAC
C2-N-3389	TCATAACTTCAGTATCATTG
C2-J-3400	ATTGGTCATCAATGATACTGA

Mcdonagh, 2009	
EF1aF	CCCATYTCCGGHTGGCACGG
EF1aR	GTCTCATGTCACGDACRGCG
Nest	GGTGGYATCGGHACAGTACC
Nest	CAGCRAMGTACCCACGACGC
NEW COIF	ATAGACGTTGAYACWCGAGC
NEW COIR	ATCCATTGCACTAATCTGCC
Nest	GTATTTGCTATTATRGCCGG
Nest	AATCCTAARAAATGTTGGGG

Otranto et al. 2005	
C2-J-2369	Simon
TL2-N-3014	Simon
GaF	GTAAACATAACATTCTTCCCCCAGCA
GaR	AAAAATGCTGGGGGAAGAATGTTA
LR-J-12887	Simon
LR-N-13398	Simon
D1	CCCCCTGAATTTAAGCATAT
D2	GTTAGACTCCTTGGTCCGTG
DGp	TGAAACTGCTTAGAATTAAGC

Lessinger and Azeredo-Espin 2000	
TM-N-193	Simon
SR-J-14233	Simon
TI-N-24	Simon
SR-J-14776	GCTGGCACGAATTTTGTC

Litjens 2001	
C1-J-2195	Simon
L2-N-3014	Simon
TM-N-193	Simon
SR-J-14233	Simon
ATP8-N-3931	AAT TGG TGC TAT TTG AGG
TW-J-1287	ACT AAT AGC CTT CAA AGC
TV-J-14122	TTC AAT GTA AAT GAA ATG
N2-N327	Oliviera 2006

Oliveira 2006	
SR-J-14941	AGCCAAAATAAAACTTA
N2-N-327	CTAAACCTATTCAAGCTCC
TI-J-42	TTACCTTGATAGGGTAAAT
TI-N-21	ATCCTATCAAGATAGCCC
N3-J-5945	CATGAATGAAATCAAGG
N5-N-6554	GGATGATCAGAATATTTTGG
N5-N-6647	GGTTCAATATGATTTATACC

C2-N-3494	GGTAAAATTACTCGATTATCAAC
C2-J-3571	AGATGTAATTCATTCTTGA
C2-N-3661	CCGCAAATTTCTGAACATTGACCA
C2-J-3696	GAAATTTGCGGGGCTAATCATAG
TK-N-3785	GTTTAAGAGACCAGTACTTG
TD-N-3862	TTTAGTTTGACATACTAATGTTAT
A8-N-3914	TCATCTAATTGGTGCTATTTGTGG
C3-J-5014	TTATTTATTTTATCAGAAGT
C3-N-5460	TCAACAAAATGTCAGTATCA
N3-J-5952	GAATGAAATCAAGGTATATTA
N5-N-6562	GATCAAGGTTGGTCAGAA
N5-J-6579	TTCTGACCAACCTTGATC
N4-N-8484	GCTAATATAGCAGCTCCTCC
N4-J-8502	GTTGGAGGAGCTGCTATATTAG
N4-N-8718	GCATATTCATCAGTTGCTCA
N4-N-8944	GGAGCTTCAACATGAGCTTT
CB-J-10612	CCAATTAATATTTCAAGATGATGAAA
CB-J-10933	TATGTTTTACCTTGAGGACAAATATC
CB-N-10920	TCCTCAAAATGATATTTGTCCTCA
CB-J-11338	CACATTCAACCAGAATGATATTT
CB-N-11328	AGCAAATAAAAAATATCATTC
CB-N-11367	ATAACTCCTCCTAATTTATTAGGAAT
CB-J-11545	ACATGAATTGGAGCTCGACCAGT
TS1-N-11683	AAATTCTATCTTATGTTTTCAAAAC
N1-N-11841	GGTACATTACCTCGGTTTCGTTATGAT
N1-N-12051	GATTTTGCTGAAGGTGAATCAGA
N1-N-12248	AAGCTAATCTAACTTCATAAG
N1-J-12314	TAGAATTAGAAGATCAACCAGC
N1-J-12533	GCATCACAAAAAGGCTGAGG
N1-J-12585	GGTCCCTTACGAATTTGAATATATCCT
N1-N-12595	GTAGCTTTTTTAACTTTATTAGAACG
LR-N-12866	ACATGATCTGAGTTCAAACCGG
LR-J-12887	CCGGTTTGAACTCAGATCATGT
LR-N-12945	GCGACCTCGATGTTGGATTAA
LR-N-13176	ACGAGAAGACCCTATAAATC
LR-J-13331	TGATTATGCTACCTTTGCACAGT
LR-J-13375	TCAGTGGGCAGGTTAGAC
LR-J-13417	ATGTTTTTGTTAAACAGGCG
LR-N-13398	CGCCTGTTTAACAAAAACAT
SR-J-14233	AAGAGCGACGGGCGATGTGT
SR-N-14275	AAGGTGGATTTGGTAGTAAA
SR-J-14508	TACAAAACAGGTTCCTCTG
SR-N-14522	ACTTAAAAAAATTTGGCGGT
SR-N-14588	AAACTAGGATTAGATACCCTATTAT
SR-J-14612	AGGGTATCTAATCCTAGTTT
SR-N-14756	GACCAAATTGGTGCCAGCAGT
SR-N-14922	AAGTTTTATTTTGGCTTA

Griffiths 2009 Microsattelites	
CheX01	TTTTATATTCTTGCTTCACA
	CAAATGAAATTGTTCAAAGT
ChEX02	GCTTCAGATTCTTGTTCAAA
	ATCCGAAGATGGTCAAAAT
ChEX03	GATCGGTTTATGTATTACCAAGA
	GCGATACTCAGTCGAGAAAA
ChEX04	GATCCTCGAGTTGACCAATA
	CCCCATATACCTGGAAGTAA

Christen 2015	
F	CGGCGATAGTGGATGTGG
CR92A1R	CGGCGATAGAATGGGAGATA
J1A2F	CGGCGATAGAATGGGAGATA
R	GCGATAGTGGATGTGGGATT

Cardoso 2016	
Cyp6g1	GAGCCCGATAAATTCGAACC
	ATCATGCCAAAACGTTCACC
foraging	TGGTGGGGTTTACAAAATCG
	TTATCCCAGCCGGTAACATC
GD	CATTCGGTGGTGCTAAGG
	CCGGGTCCAATGAAACC
Jonah65aiv	ACTGCTGCTCATTGTACTGATG
	TTGCTATTCCAGCCAGAGTG
Malvolio	TGGGGTGTGGGTATTTTAGC
	TAAAACACGACGCCAACG
PGRP-SC2	CGTGGCTGGAATGTTATGG
	TTGACCACGAGCAACAGC
RPS6-p70	TCACCAAGACGTACACCAAG
	TGTACATCCATCATTTCGTC
Smooth	TTCTTATTGCCCGATCATACAC
	AATCTTGCTCGGTGGTTG
Foraging	TCCCAAATACGATAAGGATG
	CATCACGGAATTCTTCATTG
	ACTTGCTTGGTCATAGATCG
	TTTGTAAACCCCACCAATAG
GD	AAAGGCTTTGCCTACTGCTG
	TGGTACCGTGTTCGTTCTTG
	AACGAAGCCAACTACATGAGC
	CATCACGGTAAGTGGTGAAG
Jonah65aiv	GCCTGGTGTGGTGGTTC
	CGATACCAGTATTAGATTTAATCC
Malvolio	CGTAAATTATGGGCCTTCAC
	GAATGTTTGGCATCTTCRTAC
	GTTTTTGCTCATGGCATG
	CGGCCATATGTATCACAAG
	ATGGCTGACGTGASYG

Caravalho 2009	
7F1a	AGC TAA ATC CCG AAA CTA AAC
7R4	CTG TKG ARC CNT ATC AGA C
7R3a	ATC CTT ATC ATT ATT TTC ACC C
7F4a	CTG TKG ARC CNT ATC AGA C
E3F	ATG AAT TTC AAI GTY AGY YWI ITG GAG
7R7	TTG GTT ACA CTC TAA AAT AAA TC

Cardoso 2014	
Actin	GCCATGTATGTTGCCATCC
	CGACCAGCCAAATCCAAAC
a-tub	GATTTTGACCACTCACAC
	AACGATTCAAGTTGGTG
EF1	ACAATCAGCTTGAGAGG
	ATTGATATTGCTTTGTGG
gapdh	GTCAGTGACACCCACTCCTC
	TTGATCAAGTCGATGACACG
gstD1	AAAATCAACCTGCCGATCC
	GCCAATAAAGCTAAATCGGCTA
RPL13a	CGAAGAATTGAACCTCTC
	AAGTGGAATGGACCAC
Rp49	GCACCAAGCACTTCATCC
	AGTGGGAAGCATGTGACG
RPS17	TCGTGTAAGAACCAAGAC
	GCAGATACGCTTGTTG
RPS18	TGACTTCCTCTAACTTGG
	ACCAGTAGTCTTGGTGTG
SdhA	AAGATGATTGGAAATGG
	AAGGGCATACCATAATTC

Amat 2023	
C1-J-2495	CAGCTACTTTATGAGCTTTAGG
C1-N-2800	CATTTCAAGCTGTGTAAGCATC

Alamalakala 2009	
EcoRiF	CTCGTAGACTGCGTACC
R	AATTGGTACGCAGTCTAC
MseIF	GACGATGAGTCCTGAG
R	TACTCAGGACTCAT
E	GAC TGC GTA CCA ATT C
M	GAT GAG TCC TGA GTA AC
E	GAC TGC GTA CCA ATT C NNN
M	GAT GAG TCC TGA GTA AC NN
E1	GAC TGC GTA CCA ATT C AGG
E2	GAC TGC GTA CCA ATT C ACA
E3	GAC TGC GTA CCA ATT C ACT
M1	GAT GAG TCC TGA GTA A CTA

	AAGCACCTAATGACCACC
	CGTGGTCATGGTAAAGC
RPS6-p70	TGTACATCCATCATTTCGTC

**Thyssen 2005**	
C1-j-2183	Simon paper
TL2-N-3014	
TI-N-4	
CMEG-AR	Lessinger 2004

**Lessinger 2004**	
CMEG-A	ATGATATTCTTAACTGGATT
SR-J-14776	GCTGGCACGAATTTTGTC
TI-N-24	ATTTACCCTATCAAGGTAA
CMEGAR	AATCCAGTTAAGAATATCAT
TI-N-24	
SR-J-14776	
N2-N-1128	CGTAATAATTTAATATAAAAGC
SR-J-14776	
TM-N-193	TGGGGTATGAACCCAGTAGC
CMEGAR	

**Bergamo 2015**	
7Foa	GGTATACCA TACGCCCAAC
RN2	AACAGTAATCCCTCGTACG
7FIn1	ATTGTGTCTCCCTGCAAGTG
7R1aN	CGTTTAGTTTCTGGAGCC
7FIn2	ACCATCGGTGAGTTGAGAG
7R3a	ATCCTTATCATTATTTT CACCC
380F	ACGGATT CGCG CTATTTAGA
61R	GATGACGAGCTT GTTGG TGA
1648R	CCACTCCGCCTTTAGTTTGA
2099F	TGTGCCAAAGTTGTTTCTG
1514R	GTAAG GGGTCCGTCAAAACA
2385F	CCTCGATATACAGACCGATA

**Folmer 1994**	
LCO1490	GGTCAACAAATCATAAAGATATTGG
HCO-2198	TGATTTTTTGGTCACCCTGGAGTTTA
LCO1606	TTAATTGGAGATGATCAAAT
LCO1495	ACAAATCATAAAGATATTGG
LCO1828	GGAGCTGGTACAGGTTGAAC
HCO1862	TGAACTGTTTACCCTCCTTT
HCO2042	TGATCAGTACTTATTACAGC
HCO2799	CCACGACGGTACATAGATTATGCTG
HCO2911	GAAGCTTTAGTGTGTCAACGAGGGG
HCO2192	TGATTTTTGGTCACCCTGA
HCO2798	CAACATTTTTTAGGATTAGC

M2	GAT GAG TCC TGA GTA A CTT
M3	GAT GAG TCC TGA GTA A CTG
M4	GAT GAG TCC TGA GTA A CTC
M5	GAT GAG TCC TGA GTA A CAG
M6	GAT GAG TCC TGA GTA A CAC
M7	GAT GAG TCC TGA GTA A CAA
M8	GAT GAG TCC TGA GTA A CAT

**Perdormo 2020**	
N2-J-1480	TACAATTTATCGCCTAAACTTCAGCC
C2-N-3793	GAGACCATTACTTGCTTTCAGTCATCT
C2-J-3662	GGTCAATGTTCAGAAATTTGTGG
N3-N-5942	TGATTTCATTCATGATATAAGTCC
N5-J-7660	TTCTGATCATCCCTGATC
N5-N-8853	GTAAAATCTTATAATGCTGG
N5-J-8621	GCTATAGCAGCTGGCAATCAAG
N4-N-9676	CTTATGAACGGATGGGGAGTC
N4-J-9580	TAGGAGGAGCAGCCATATTTGGC
NL-N-10740	GAAGGTCTTGGGCTTTCT
NL-J-10725	AAGCCCAAGACCTTC
CB-N-11872	GATGCACCATTGGCATGT

**Yusseff-Vanegas 2016**	
LCO1490	GGTCAACAAATCATAAAGATATTGG
CI-N-2776	GGATAATCAGAATATCGTCGAGG
B1	CCCATYTCCGGHTGGCACGG
C1	CTCTCATGTCACGDACRGCG
D1.F	CCCCCTGAATTTAAGCATAT
D35.486.R	TCGGAAGGAACCAGCTACTA
ITS4	TCCTCCGCTTATTGATATGC
ITS5.8	GGGACGATGAAGAACGCAGC

**Fresia 2011**	
CRc-N-BVII-R	Duarte 2008
SR-J-14875	AAGTCACACAAAAATTTAC
TW-J-1287	Lyra 2009
C1-N-2320	AATCCTAATAATCCAATAGC
TL2-J-3034	Simon paper
TK-N-3785	

**Duarte 2008**	
C1-N-1560	Simon Paper
SR-J-14612	
TM-N-193	
Crc-J-BVII	TTTTATATTGAAGCGTCG
Crc-N-BVII R	TTGCACGGTTCAATATAA
CMegAR	Lessinger 2004
CMegAR	

Malgorn 1999	
F1	TTA TCA TTA CCA GTA TTA GC
R1	AGC TGA AGT AAA GTA AGC T
F2	CTA CTT TAT GAG CTT TAG G
R2	TTC AAG TTG TGT AAG CAT C

Newcomb 1997	
7F1a	AGCTAAATCCCGAAACTAAAC

Singh 2012	
Calcad207F	GAC CAT TTG TTT TGG CTG CT
Calcad908R	AAT CAA TGG ATT GGG GAG AA
Calcad627F	AGT TCG ATT GGT GTG CTG TG
Calcad1011R	CAG AAA GCT ATG GCC GAT TC
Oescado300F	AAC CCA CCG ATA AGA GAC CA
Oescado594F	TCG ATA CTG TGG CTG GTG AA
Oescado770F	TTG AGA AAT CTG GGC AAA CC
Oescado1287R	TCC TTG GCT TCC GTT AAG AA
CadT2207F	AGT GTG GGC GAG GTA ATG TC
CadT2370F	CTG CCT TGA AAG CCA ACT TC
CadT3156R	CAG AAA GCT ATG GCC GAC TC

Torres 2005	
CH21	GGCGAGCGAGTCCTATTAC
R	CAGTTCCAGGTCAATTTCC
CH23	GTTAAGAATATCCTGCTAATG
R	TTTATATGTCCCATTGTGC
CH24	CCCACCAAAAACAATGTC
R	GTTTCCCACCAGTTCAGG
CH25	CACCTACCTTAAGTGACACG
R	TTTTTCCTCTTCTGACCTC
CH26	TATTTTCCCTTCATTCTTG
R	GGTGTTTTAATACCCGTAAAC
CH28	CAATCACCAACCAAAATG
R	TGTATAAAGATGCGATTGAC
CH29	CATCTACGGTGGTGTAGG
R	ACATGATATTTTCGCTCTC

Wells 2001	
TY-J-1460	TACAATTTATCGCCTAAACTTCAGCC
C1-N-1687	CAATTTCCAAATCCTCCAATTAT
C1-J-1751	GGATCACCTGATATAGCATTCCC
C1-N-1840	AGGAGGATAAACAGTTCAC/TCC
C1-J-2183	CAACATTTATTTTGATTTTTTGG
C1-N-2191	CCCGGTAAAATTAAAATATAAACTTC
C1-J-2319	TAGCTATTGGAC/TTATTAGG
C1-N-2293	AGTAAACCAATTGCTAGTATAGC

CRv-J-Cadu	TTGGTAAATCCTACTGT
SR-J-14941	Oliviera 2006

McDonagh 2009b	
COX1F1	ATAGACGTTGAYACWCGAGC
COX1R1	ATCCATTGCACTAATCTGCC
COX1IntF1	GTATTTGCTATTATRGCCGG
CoX1 intR4	AATCCTAARAAATGTTGGGG
B1	CCCATYTCCGGHTGGCACGG
C1	GTCTCATGTCACGDACRGCG
Fint	GGTGGYATCGGHACAGTACC
Rint	AGTTTCRACACGACCGACG
EF1	ACAGCGACGGTTTGTCTCATGTC
EF2	CACATTAACATTGTCGTGATTGG
EF3	CCGATACCACCGATTTTGTA
EF4	CCTGGTTCAAGGGATGGAA
C1-J1751a	GGATCACCTGATATAGCATTCCC
C1-J-2183	CAACATTTATTTTGATTTTTTGG
C1-J-2495	CAGCTACTTTATGAGCTTTAGG
C1-N-1840	AGGAGGATAAACAGTTCAC/TCC
C1-N-2191	CCCGGTAAAATTAAAATATAAACTTC
C1-N-2659	GCTAATCCAGTGAATAATGG
TL2-N-3013	TCCATTACATATAATCTGCCATATTAG
TY-J-1460	TACAATTTATCGCCTAAACTTCAGCC
UEA7	TACAGTTGGAATAGACGTTGATAC
UEA10	TCCAATGCACTAATCTGCCATATTA
D1.F	CCCCCTGAATTTAAGCATAT
D2.R	GTTAGACTCCTTGGTCCGTG
D1.R	CTCTCTATTCAGAGTTCTTTTC
D2.F	GAGGGAAAGTTGAAAAGAAC
D3	GACCCGTCTTGAAACACGG
D7.R	CGACTTCCCTTACCTACAT
D3-5.R	TTACACACTCCTTAGCGGA
D35.486.R	TCGGAAGGAACCAGCTACTA
D35.742.F	TCTCAAACTTTAAATGG
D7.F	GACTGAAGTGGAGAAGGGT
SR-J-14233	AAGAGCGACGGGCGATGTGT
SR-J-14776	GCTGGCACGAATTTTGTC
TI-N-24	ATTTACCCTATCAAGGTAA
TM-N-193	TGGGGTATGAACCCAGTAGC

Yusseff-Vanegas 2017	
Primer 1	TACAATTTATCGCCTAAACTTCAGCC
C1-N-2191	CCCGGTAAAATTAAAATATAAACTTC
C1-J-1751	GGAGCTCCTGACATAGCATTCCC
C2-N-3014	TCCATTGCACTAATCTGCCATATTA

C1-J-2495	CAGCTACTTTATGAGCTTTAGG
C1-N-2514	AACTCCAGTTAATCCTCCTAC
C1-N-2659	GCTAATCCAGTGAATAATGG
C1-J-2792	ATACCTCGACGTTATTCAGA
C1-N-2800	CATTTCAAGT/CTGTGTAAGCATC
TL2-N-3014	TCCAATGCACTAATCTGCCATATTA
C2-J-3138	AGAGCCTCTCCTTTAATAGAACA
C2-N-3389	TCATAAGTTCA[R]TATCATTG
C2-J-3408	CAATGATAT/CTGAAGT/ATATGA
TK-N-3775	GAGACCATTACTTGCTTTCAGTCATCT

**Sperling 1994**	
a	CAACATTTATTTTGATTTTTTGG
b	GAGACCATTACTTGCTTTCAGTCATCT
a	CAACATTTATTTTGATTTTTTGG
c	CTAATCCAGTGAATAATGG

**Friedrich and Tautz 1997**	
687	CGG TGG ATC ACT (C/T)GG
427	CCC(C/G)CGTAA(T/C)TTAAGCATAT
686	CTCTCTATTCA(A/G)AGTTCTTT(G/C)
427	
485	ACCCGTCTTGAAACACGA
689	ACACACTCCTTAGCGGA
476	CTGAAGTGGAGAAGGGT
477	GACTTCCCTTACCTACAT
486	TCGGAAGGAACCAGCTACTA
706	GCCAGTTCTGCTTACC
742	CTCAAACTTTAAATGG
487	AGGGTTTCGTGTGAACAG
703	TTCCAAACC(A/C)TATCTC
743	CGATTTTCAAGGTCC

**Parsons 2000**	
L14816	CCATCCAACATCTCAGCATGATGAAA
H15173	CCCCTCAGAATGATATTTGTCCTCA

**Bogdanowicz 1993**	
S1751	GGATCACCTGATATAGCATTCCC
S1859	GGAACIGGATGAACWGTTTAYCCICC
S2792	ATACCTCGACGTTATTCAGA
A2191	CCCGGTAAAATTAAAATATAAACTTC
A3014	TCCAATGCACTAATCTGCCATATTA
A3389	TCATAAGTTCARTATCATTG
A3772	GAGACCATTACTTGCTTTCAGTCATCT

**Lunt 1996**	
UEAl	GAATAATTCCCATAAATAGATTTACA
UEAZ	TCAAGATAAAGGAGGATAAACAGTTC
UEAl	TATAGCATTCCCACGAATAAATAA
UEA4	AATTTCGGTCAGTTAATAATATAG
UEA5	AGTTTTAGCAGGAGCAATTACTAT
UEA6	TTAATWCCWGTWGGNACNGCAATRATTAT
UEA7	TACAGTTGGAATAGACGTTGATAC
UEA8	AAAAATGTTGAGGGAAAAATGTTA
UEA9	GTAAACCTAACATTTTTTCCTCAACA
UEA10	TCCAATGCACTAATCTGCCATATTA

**Stevens and Wall 2001**	
D1F	CCCCCTGAATTTAAGCATAT
D1R	GTTAGACTCCTTGGTCCGTG
D1R	CTCTCTATTCAGAGTTCTTTTC
D2F	GAGGGAAAGTTGAAAAGAAC
D3-5F	GACCCGTCTTGAAACACGG
D7R	CGACTTCCCTTACCTACAT
D3-5R	TTACACACTCCTTAGCGGA
D3-5.486R	TCGGAAGGAACCAGCTACTA
D3-5.742F	TCTCAAACTTTAAATGG
D7F	GACTGAAGTGGAGAAGGGT

**Zhang 1997**		
UEA1	Ty-N-1438	GAATAATTCCCATAAATAGAACA
UEA1d	TY-N-1438d	GAAWAATTCCYATAAWTARATTTACA
UEA2	C1-N-1844	TCAAGATAAAGGAGGATAAACAGTTC
UEA2d	C1-N-1843d	GMWARWGGWGGRTAWACWGTTCA
UEA3	C1-J-1763	TATAGCATTCCCACGAATAAATAA
UEA4	C1-N-2087	AATTTCGGTCAGTTAATAATATAG
UEA4d	C1-N-2096d	GANGTATTWARRTTTCGRTCWGTTA
UEA5	C1-J-2090	AGTTTTAGCAGGAGCAATTACTAT
UEA6	C1-N-2395d	TTAATWCCWGTWGGNACNGCAATRATTA

UEA7	C1-J-2369	TACAGTTGGAATAGACGTTGATAC
UEA8	C1-N-2735	AAAAATGTTGAGGGAAAAATGTTA
UEA9	C1-J-2753	GTAAACCTAACATTTTTTCCTCAACA
UEA10	TL2-N-3014	TCCAATGCACTAATCTGCCATATTA

Supplemental Table 3. Real-time PCR Master Mix

Reagent	Initial Concentration (µM)	Final Concentration (nM)	Volume per Reaction (µl)
Nuclease-free Water	NA	NA	10.25
TaqMan Fast Virus	4	1000	6.25
NWS Forward Primer	20	750	0.9375
NWS Reverse Primer	20	750	0.9375
Xeno Liz Probe	10	400	1
NSW Probe	10	250	0.625
$MgCL_2$	25	1000	1
Template	NA	NA	5
		Total Volume	25

Supplemental Table 4: Real-time PCR Cycling Conditions

Cycles	Block 1: NWS1	Block 2: NWS4	Block 3: NWS5	Block 4: NWS10	Block 5: NWS12
**1**	95°C | 2min	--	--	--	--
**40**	95°C | 30sec<br>52°C | 30sec<br>62°C | 1:30min	54°C | 30sec<br>62°C | 1min	57°C | 30sec<br>62°C | 1:45min	56°C | 30sec<br>62°C | 2:30min	54°C | 30sec<br>62°C | 1:20min
**1**	62°C | 7min	--	--	--	--

Supplemental Table 5. Sanger Master Mix

Reagent	Volume (µl)	Final Concentration
H_2O	11.55	--
10xBFR	2.5	1X
DMSO	1.25	5%
$MgCl_2$	2.5	2.5nM
dNTP	2	0.2nM
Primers	2	0.4µM
Taq	0.2	1U/rxn
BSA	1	4%
DNA	2	--
